# Comparison of methods to identify long term care nursing home residence with administrative data

**DOI:** 10.1186/s12913-017-2318-9

**Published:** 2017-05-30

**Authors:** James S. Goodwin, Shuang Li, Jie Zhou, James E. Graham, Amol Karmarkar, Kenneth Ottenbacher

**Affiliations:** 1 Department of Medicine, University of Texas Medical Branch, 301 University Boulevard, Galveston, TX 77555 USA; 2 Sealy Center on Aging, University of Texas Medical Branch, 301 University Boulevard, Galveston, TX 77555 USA; 3 Division of Rehabilitation Sciences, University of Texas Medical Branch, 301 University Boulevard, Galveston, TX 77555 USA; 4 George and Cynthia Mitchell Distinguished Chair in Geriatric Medicine, University of Texas Medical Branch, 301 University Blvd., Galveston, TX 77555-0177 USA

**Keywords:** Nursing home, Long term care, Minimum Data Set, Medicare

## Abstract

**Background:**

To compare different methods for identifying a long term care (LTC) nursing home stay, distinct from stays in skilled nursing facilities (SNFs), to the method currently used by the Center for Medicare and Medicaid Services (CMS). We used national and Texas Medicare claims, Minimum Data Set (MDS), and Texas Medicaid data from 2011-2013.

**Methods:**

We used Medicare Part A and B and MDS data either alone or in combination to identify LTC nursing home stays by three methods. One method used Medicare Part A and B data; one method used Medicare Part A and MDS data; and the current CMS method used MDS data alone. We validated each method against Texas 2011 Medicare-Medicaid linked data for those with dual eligibility.

**Results:**

Using Medicaid data as a gold standard, all three methods had sensitivities > 92% to identify LTC nursing home stays of more than 100 days in duration. The positive predictive value (PPV) of the method that used both MDS and Medicare Part A data was 84.65% compared to 78.71% for the CMS method and 66.45% for the method using Part A and B Medicare. When the patient population was limited to those who also had a SNF stay, the PPV for identifying LTC nursing home was highest for the method using Medicare plus MDS data (88.1%).

**Conclusions:**

Using both Medicare and MDS data to identify LTC stays will lead to more accurate attribution of CMS nursing home quality indicators.

**Electronic supplementary material:**

The online version of this article (doi:10.1186/s12913-017-2318-9) contains supplementary material, which is available to authorized users.

## Background

There has been an increasing interest in studying the care provided in nursing homes, stimulated by clear evidence of variation in utilization, quality of care, outcomes, and cost [1–5]. However, there are several barriers to conducting national studies on these issues. One important barrier is the availability of a robust method for differentiating residents with a long term care (LTC) stay from those with a skilled nursing facility (SNF) stay. SNFs typically provide rehabilitation nursing services and medical care for short stay residents immediately following hospitalization. SNF is covered 100% by Medicare Part A for eligible patients for the first 20 days following hospitalization, and 80% for days 21–100 [3]. Community discharge is the primary objective of SNFs, and is a quality measure identified by the Centers for Medicare and Medicaid Services (CMS). LTC services are often custodial care, and are covered by Medicaid, private pay, or LTC insurance [6].

The Omnibus Budget Reconciliation Act (OBRA) of 1987 mandated that all nursing homes receiving Medicare or Medicaid payments complete a standardized assessment of the physical, cognitive, emotional, and functional health of their residents, resulting in the Minimum Data Set (MDS) [7]. However, MDS does not have a specific variable differentiating LTC residents from those in a SNF. Most nursing facilities provide both SNF and LTC services, and patients can transition from SNF to LTC while retaining the same room and bed. Previous studies have developed algorithms using administrative claims data to identify nursing home stays [8–13] and to differentiate LTC residents from those with a SNF stay [8, 11, 12].

The current CMS quality reporting program for nursing homes uses length of stay to differentiate short stays (≤100 days) from long stays (> 100 days), and uses only MDS data [14]. This method can conflate services in a SNF with services in a LTC bed. For example, a patient who is discharged from a hospital to SNF for a 20 day stay, followed by a 2 month stay in a LTC bed at the same facility, is classified as short stay. Quality measures generated for this stay are attributed to SNF care. Conversely, quality measures related to a patient who spends 80 days in a SNF followed by 40 days in LTC would be attributed solely to the LTC services. Obviously, fair quality measures are important for both SNF and LTC settings, which depend on accurate differentiation between the two sites.

For this study, we compared the performance of two methods using combinations of Medicare Part A and B claims and MDS to the method currently used by CMS.

## Methods

### Data sources

We used Medicare, Medicaid, and MDS data in the analyses. Most of the validation analyses were performed with linked Medicare-Medicaid data from Texas. We selected all beneficiaries who were enrolled in full Medicare Parts A and B and had full Medicaid coverage in Texas from 1/1/11 to 12/31/11 or date of death (*n* = 575,472). We also constructed another cohort consisting of the 9,022 enrollees in the cohort above who had a SNF stay within 3 days of hospital discharge between 01/01/2011 and 6/30/2011. This cohort also had full Medicare Part A and B, no health maintenance organization (HMO) in 2011, and no nursing facility service within 3 months prior to hospital discharge. We followed each patient in this cohort 180 days after SNF admission. We used Medicaid charges for LTC Services as a gold standard to assess the sensitivity and positive predictive value (PPV) of the other measures, similar to other investigators [12].

### Ethics

The University of Texas Medical Branch Institutional Review Board approved this study.

### Methods to identify LTC nursing home stays

1. Method using Part A and B claims data
  This method was based on the method of Yun et al. [12]. We first used Medicare Part A SNF claims to identify the dates billed for SNF services [8, 12]. These were used to exclude SNF stays from the potential LTC stays identified by Part B claims. Part B claims data contain two types of indicators for care provided in a nursing home. Both sets of codes are used regardless of whether the patient is receiving SNF or LTC services. The first are Evaluation and Management (E&M) charge codes in either the carrier or outpatient file for professional services provided in a nursing home: 99304-99310, 99315-99316, or 99318. Second, the carrier file also includes place-of-service codes specifically for nursing homes: 32 or 33. Any of these codes for dates outside a SNF stay identified in the Part A data were classified as LTC stays. This method cannot determine admission or discharge dates or length of stay in a LTC nursing home.
2. Method using Part A plus MDS data
  This method was based on the method described by Intrator et al. [8] The content of the MDS assessment is the same for SNF and LTC patients, with differences in frequency of assessment [14, 15]. We defined an MDS episode based on the CMS method, which starts with the entry date (variable name: A1600_ENTRY_DT) and ends with the date of the last MDS assessment (variable name: TARGET_DATE) that lists that same entry date. We then excluded SNF stays from the MDS episode as in method 1. MDS episodes with more than 100 days duration outside SNF stays were then classified as LTC episodes. We also performed sensitivity analyses using more than 30 days outside of SNF stays.
3. The CMS method, using MDS data alone
  The current method used by CMS to differentiate SNF versus LTC services uses only MDS data. All MDS episodes >100 days in a nursing facility are termed “long stay,” and all episodes ≤ 100 days are short stay. A given nursing facility episode can be interrupted by periods outside the nursing facility, such as a hospitalization, as long as the patient returns to the nursing faculty afterward. The days outside of the facility are not counted in determining length of stay [14].

### Validation using Medicaid data

We determined the extent of agreement for each of the three methods of identifying a LTC stay with the gold standard of a LTC nursing home stay identified in the Medicaid data. Medicaid is the primary payor for approximately 70% of LTC nursing home services [16]. Medicaid might also provide the co-pay for a SNF stay beyond 20 days. To account for these cases, we determined a Medicaid LTC stay after excluding dates of SNF stays determined from the Medicare Part A claims. We then defined a LTC nursing home stay as more than 100 consecutive days of Medicaid claims for nursing home care that were not also in Part A claims. We also used a cut off of more than 30 days of Medicaid claims for LTC services in order to assess the impact of length of stay on the sensitivity of the different methods to identify LTC stays.

We calculated sensitivity as the percentage of LTC episodes identified in Medicaid which overlap with a LTC episode identified by each of the three methods. We calculated PPV as the percentage of LTC episodes identified by each method which overlaps with a LTC episode identified by Medicaid. Both sensitivity and PPV were estimated using the cohort of 575,472 beneficiaries who had both full Medicare Parts A and B and full Medicaid coverage in 2011 (see Data Source, above). Method 1, which relies on E&M charges and place-of-service codes, does not provide information on the beginning or end dates of a LTC episode. Thus, the sensitivity of this method is the percentage of Medicaid LTC episodes which contain at least one relevant E&M charge or place-of-service code, and the PPV is the percentage of patients with E&M or place-of-service charges where at least one such charge occurs within a LTC nursing home episode as defined by Medicaid data. Because we cannot distinguish between 30 and 100 day lengths of stay with method 1, we calculate only one PPV for method 1.

The sensitivity and PPV were estimated based on at least one day of overlap between the LTC episodes identified by the different methods and the LTC episodes identified by Medicaid. We also explored the effect on sensitivity and PPV of increasing the days of overlap required for a true positive from 1 to 100.

We constructed a Venn diagram to illustrate the overlap of the three methods with the gold standard of Medicaid data, using R software (www.r-project.org) version 3.3.2. An overlap was identified if two or more methods identified episodes with at least one day in common in LTC.

### Estimating rate of LTC nursing home residence nationally

We used the three methods to estimate the percentage of older adults who spent more than 100 days in a LTC nursing home in each of the 50 states in 2012. For method 1, we used a 5% sample of National Medicare Part A and B data. For method 2 we used 100% national Medicare Part A data linked to the MDS. For method 3, we used 100% national MDS data. We examined the correlations among the different methods in their estimates of state rates of LTC, and we also correlated those rates with the rates of LTC nursing home residents in 2012 produced by the National Study of Long-Term Care Providers [17], using Pearson correlations.

## Results

Table 1 summarizes the characteristics of the 575,472 patients in the cohort who had Medicare-Medicaid dual eligibility in 2011. This population had large minority representation, with 34% Hispanic and 18% African American, and a high number of comorbidities. Approximately 8.1% experienced an episode of more than 100 days in a LTC facility in 2011, as determined by Medicaid data. We then used the determination of a LTC episode in Medicaid as the gold standard to validate the three methods of identifying a LTC stay. These results are summarized in Table 2. For lengths of stay > 100 days, the sensitivity of all methods was > 92%. The PPV was 84.6% for method 2 which used Medicare Part A plus MDS data, 66.5% for the method that used Medicare Part A and B alone (method 1) and 78.7% for the CMS method which used MDS alone (method 3).

**Table 1:** Description of patients dually eligible for Medicare and Medicaid used in the validation, in Texas 2011

Patient characteristics	Cohort 1	Cohort 2
Overall	N = 575,472	N = 9,022
Age		
< 65	201,063 (34.94%)	1,923 (21.31%)
> =65, <75	172,810 (30.03%)	2,360 (26.16%)
> =75, <85	136,135 (23.66%)	2,818 (31.23%)
> =85	65,464 (11.38)	1,921 (21.29%)
Race		
White	216,337 (37.59%)	4,287 (47.52%)
Black	102,160 (17.75%)	1,692 (18.75%)
Hispanic	194,297 (33.76%)	2,312 (25.62%)
Others	62,678 (10.98%)	731 (8.10%)
Sex		
Female	364,833 (63.40%)	6,094 (67.55%)
Male	210,639 (36.60%)	2,928 (32.45%)
Location		
Metropolitan	458,433 (79.70%)	6,867 (76.11%)
Non-Metropolitan	116, 839 (20.30%)	2,155 (23.89%)
Number of comorbidities		
0,1	172,746 (30.02%)	30 (0.33%)
2,3	123,579 (21.47%)	226 (2.50%)
4,5	102,699 (17.85%)	673 (7.46%)
6,7	97,958 (17.02%)	2,057 (22.80%)
> =8	78490 (13.64%)	6,036 (66.90%)
Resident in LTC 100 days in 2011		
Yes	46,451 (8.07%)	317 (3.51%)
No	529,021 (91.93%)	8,705 (96.49%)

*LTC* long term care

**Table 2:** Validation of different methods of identifying a long term care (LTC) nursing home stay using Medicare and MDS data, vs. data in Medicaid as the gold standard, in patients dually eligible for Medicare and Medicaid

Method	LTC nursing home length of stay in Medicaid data	
	Cut off^a^ Length of stay >30 days	Cut off^a^ Length of stay >100 days
	N = 84,869	N = 53,837
1) Part A and B with E&M Charge for NH Services		
Sensitivity	89.68%	92.55%
PPV^b^		66.45%^b^
2) Part A with MDS episode of care		
Sensitivity	93.96%	93.09%
PPV	91.60%	84.65%
3) CMS Method: MDS alone		
Sensitivity	77.49%	93.64%
PPV^b^		78.71%^b^

*E&M* evaluation and management, *NH* nursing home, *PPV* positive predictive value, *MDS* Minimum Data Set, *CMS* Centers for Medicare and Medicaid Services

^a^For cut off of > 30 or >100 day lengths of stay, the Medicaid data demonstrated > 30, and > 100 day length of stay in the validation

^b^Only one PPV could be detected for the different lengths of stay in methods one and three. Method one relies on provider charges to identify nursing home stays, and length of stay cannot be reliably estimated. Method three uses any length of stay in a nursing facility (whether skilled nursing facility or LTC) > 100 days as the criterion

**Fig. 1:**
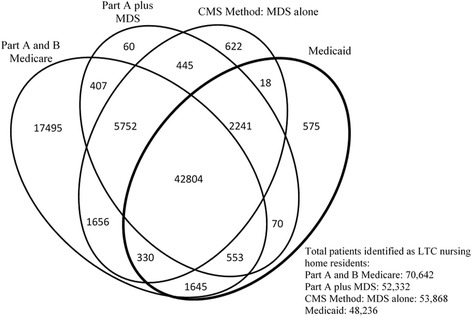
Venn diagram illustrating overlap of the three methods with each other and with the gold standard of long term care (LTC) stays identified in Medicaid. Please note that the areas shown in the figure are not proportional to the numbers in each category. These analyses were done at the level of enrollee, determining whether the individual enrollee resided in a LTC nursing home for > 100 days in 2011. The analyses presented in Tables 2 and 3 are conducted at the episode level, comparing episodes in a LTC nursing home identified by the different methods. The results are similar with the two approaches

Figure 1 presents a Venn diagram showing the overlap in identification of patients with a LTC nursing home stay of more than 100 days among the three methods, and the results from Medicaid data. Medicaid data identified 46,451 patients in LTC in 2012. The total number of patients identified in LTC using methods 1, 2, and 3 were 70,642, 51,017, and 53,868, respectively.

Also shown in Table 2 are the validation results for identifying LTC stays of > 30 days rather than of > 100 days. The sensitivity for method 2 increased slightly to 94.0%, while it decreased to 89.7% for method 1 and to 77.5% for method 3. The PPV for method 2 was 91.6% to identify a LTC stay of > 30 days versus a 84.7% PPV for LTC stays of > 100 days. We could not estimate a PPV for methods 1 and 3 because method 1 cannot estimate length of stay and method 3 defines a LTC stay as > 100 days length of stay.

**Fig. 2:**
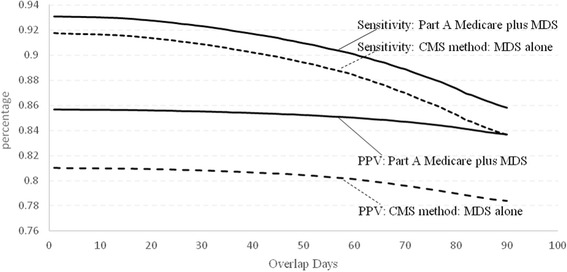
Sensitivity and positive predictive value (PPV) of methods 2 and 3 for identifying a long term care (LTC) stay, as a function of the number of days identified by each method that are also identified with Medicaid data. The sensitivities and PPVs shown in Table 2 were generated with the rule that there was at least one day of overlap between the LTC episode identified by a method and an episode identified using Medicaid claims. The sensitivities of both methods decline as the number of days of required overlap with Medicaid increases. There are smaller declines in the PPVs

In Table 2, a LTC nursing home stay identified by each of the methods is called a true positive if there is at least one day of overlap between the dates in LTC identified by Medicaid data and those identified by the method. We next explored the amount of overlap of dates identified by methods 2 and 3 and the dates in LTC indicated in Medicaid data. With method 2 (Part A plus MDS), the median overlap with Medicaid data was 94.6% days, compared to 87.5% days for method 3 (CMS method). Method 1, based on a provider charge for nursing home services, does not allow for determination of admission and discharge dates from LTC. The PPVs for each method declined slightly as the number of days of overlap required increases from one to 100, while the sensitivities showed a steeper decrease (Fig. 2).

We also examined whether the sensitivity and PPV of the methods varied by patient characteristics. We stratified patients by gender, age, race/ethnicity, hospitalization in 2011, SNF stay in 2011, and the individual’s residential location. The only major differences were by whether the patient had been in a hospital or SNF during that year (Additional file 1). To further explore that relationship, we tested the three methods with a dual eligible cohort of patients who were hospitalized and then discharged to a SNF. This is a more rigorous test of whether the methods correctly identify those who are in a LTC bed vs. a SNF bed, because all of these patients were initially discharged to a SNF. The results are shown in Table 3. The sensitivity of the method to identify LTC stays that lasted more than100 days was highest (92.4%) for method 1. The other two methods had sensitivities of approximately 80%. The PPV for method 1 was only 64.4%, versus 88% for method 2 and 72.7% for the CMS method (method 3).

**Table 3:** Validation of different methods of identifying a long term care (LTC) nursing home stay using Medicare and MDS data, vs. data in Medicaid as the gold standard, restricted to patients who were hospitalized and discharged to a skilled nursing facility

Method	LTC nursing home length of stay in Medicaid data	
	Cut off Length of stay >30^a^	Cut off Length of stay >100^a^
	N = 1,666	N = 317
1) Part A and B with E&M Charge for NH Services		
Sensitivity	88.79%	92.42%
PPV^b^		64.59%^b^
2) Part A with MDS episode of care		
Sensitivity	85.05%	78.86%
PPV	87.38%	88.07%
3) CMS Method: MDS alone		
Sensitivity	69.39%	80.12%
PPV^b^		72.70%^b^

*MDS* minimum data set, *E&M* evaluation and management, *NH* nursing home, *PPV* positive predictive value, *CMS* Centers for Medicare and Medicaid Services

^a^For the cut off of > 30 or >100 day lengths of stay, the Medicaid data demonstrated > 30 and > 100 day length of stay in the validation

^b^Only one PPV could be detected for the different lengths of stay in methods one and three. Method one relies on a provider charges to identify nursing home stays, and length of stay cannot be reliably estimated. Method three uses any length of stay in a nursing facility (whether skilled nursing facility or LTC) > 100 days as the criterion

Finally, we used each of the three methods to estimate the percent of Part A and B Medicare recipients age 65 and over who were in a LTC bed for more than 100 days in 2012 in each of the 50 states, and compared those estimates to results from the National Study of Long-Term Care Providers in 2012 [17]. The state rates generated by methods 2 and 3 were highly intercorrelated (r ≥ 0.98), and were also each highly correlated with the estimated rates from the National Study of Long-Term Care provider’s survey, with r ≥ 0.96. The rates produced by method 1 demonstrated relatively weaker associations with the other two methods (r > 0.82) and with the survey estimates (r = 0.82).

## Discussion

We compared three methods for identifying a LTC stay, and for differentiating these stays from SNF stays. There are several general conclusions that can be drawn from the analyses presented. First, all methods had acceptable sensitivities (˃92%) in identifying a stay in a LTC bed of more than 100 days duration. Second, the method using Medicare Part A and B data produced many more false positives than the other methods, with a lower PPV. Third, the sensitivities of methods 1 and 3 were lower when all LTC nursing stays of > 30 days were included. This is especially true for method 3, the current method employed by CMS to distinguish SNF from LTC stay nursing home episodes. For method 3, the sensitivity for identifying of all long term care episodes > 30 days was only 77%. The data from the rest of those episodes would erroneously contribute to the quality measures for the SNF beds.

The sensitivities and PPVs for the three methods were lower when applied to a validation cohort that included only patients hospitalized and discharged to a SNF. Most new admissions to LTC are preceded by a SNF stay [18]. Because 100% of this validation cohort started in a SNF, this was a more rigorous test of how well the methods could differentiate a stay in a LTC bed vs. a SNF bed. In particular, method 3, the current method used by CMS, had a PPV of only 72.7%. Thus, many of the “long stay” episodes identified by this method would be incorrectly used in generating quality measures of LTC facilities.

The three methods differ in the data sets required. Method 1 uses only Medicare charge data from Parts A and B while Method 3 uses only MDS. Method 2 require both Medicare and MDS data, and can lose patients in the data linkage process. Approximately 3% of patients in the Medicare- Medicaid dual eligible cohort with a LTC episode in Medicaid could not be linked to MDS. This is reflected in the sensitivity of that method. The method using Part A and B Medicare data had good sensitivity but poor specificity. Physicians and other providers may use the E&M charge for services provided in a nursing facility to bill for services provided in other institutional settings such as group homes or assisted living. In any case, this method produced many more false positives than did the methods using Part A Medicare data plus MDS or MDS data alone.

The importance of our findings are directly related to the implementation of patient-centered, value-based outcomes as described in the Affordable Care Act and the Improving Medicare Post-Acute Care Transformation (IMPACT) Act [19–21]. CMS recently introduced six new quality metrics to its consumer-based Nursing Home Compare website [22]. Four of the six (discharge to the community, emergency department visits, re-hospitalization, and improvement in function) are related to “short-stay” (SNF) patients. Two quality measures (decrease in ability to move independently and receipt of anti-anxiety or hypnotic medications) are for LTC residents. The CMS determination of short-stay vs. long-stay is based on Method 3 described above, i.e., long-stay is an episode > 100 days based on MDS data. As noted previously, there are potential inaccuracies in identifying patients as LTC using only duration of the episode. Misclassification or failure to identify patients correctly by setting and/or services received has clear implications for the validity and usefulness of new and existing quality measures.

Part of the problem in distinguishing between SNF and LTC nursing home services stems from the vocabulary used. “Long term care” implies an extended stay. This is the rationale behind distinguishing SNF from LTC by a cut-off in length of stay. However, SNF and LTC beds provide very different services: rehabilitation vs custodial care. And as noted in the introduction, defining services only by length of stay, rather than the actual services provided, can lead to inaccurate attribution of CMS quality ratings.

Of particular relevance to our findings is the fact that three of the new CMS quality measures in Nursing Home Compare are based on Medicare Part A claims data [22]. This is significant because it represents the first time CMS has included quality measures in Nursing Home Compare that are not based solely on information from the MDS. Our findings suggest that including a combination of Medicare Part A claims and MDS information identifies LTC stays with higher sensitivity and PPV than the approach currently used by CMS.

There are several limitations associated with this study. First, the methods using Medicare Parts A and B (method 1) and MDS plus Part A Medicare are only relevant for nursing home residents with Medicare. Some quality measures are obtained from sources other than MDS, such as the results of inspections. Length of stay might be the only way to distinguish SNF from LTC for patients not enrolled in Medicare. Second, the Medicaid validation was performed with Texas data only, due to a data availability issue, which may not be generalizable. However, the sources of the data used in the three methods, Medicare and the MDS, are federally mandated and monitored, which would reduce but not eliminate any geographic variations in data quality. In addition, the high correlation between the estimates of LTC nursing home residence by state between the methods and data obtained by the CDC nursing home survey provides indirect support for the generalizability of the validation [17]. Another limitation is that the gold standard in the validation cohorts was Medicaid data. Medicaid is the payor for approximately 70% of LTC nursing home residents [16]. The validation did not include residents for whom the LTC stays were reimbursed by means other than Medicaid. This limitation may contribute to an underestimation of the true PPV of these methods.

## Conclusions

In summary, all three methods had acceptable sensitivities in identifying individuals who resided in a LTC bed for more than 100 days, but the method based on Medicare Part A and B data had a low PPV. In analyses including LTC nursing home stays of more than 30 days, or in a population that had at least one SNF stay, the method combining Medicare Part A with MDS data had the best performance. CMS should consider adopting that method in order to more accurately attribute its quality ratings to the correct services.

## Additional file

Additional file 1: Table S1A. Validation of three methods of identifying a LTC nursing home stay using Medicaid charges for LTC services as the gold standard, in patients dually eligible for Medicare and Medicaid, stratified by age. **Table S1B.** Validation of three methods of identifying a LTC nursing home stay using Medicaid charges for LTC services as the gold standard in patients dually eligible for Medicare and Medicaid, stratified by race. **Table S1C.** Validation of three methods of identifying a LTC nursing home stay using Medicaid charges for LTC services, as the gold standard in patients dually eligible for Medicare and Medicaid, stratified by hospitalization or SNF stay in that year. **Table S1D.** Validation of three methods of identifying a LTC nursing home stay using Medicaid charges for LTC services as the gold standard in patients dually eligible for Medicare and Medicaid, stratified by gender and location. (DOCX 16 kb)

## Abbreviations

CMS: the Centers for Medicare and Medicaid Services
E&M: Evaluation and management
HMO: Health maintenance organization
IMPACT: Improving Medicare Post-Acute Care Transformation
LTC: Long term care
MDS: Minimum data set
OBRA: The Omnibus Budget Reconciliation Act of 1987
PPV: Positive predictive value
SNF: Skilled nursing facility
