# Supplementary material for: Comparison of methods to identify long term care nursing home residence with administrative data
Source: BMC Health Serv Res. 2017 May 30;17:376. doi: 10.1186/s12913-017-2318-9 (PMC5450097; doi:10.1186/s12913-017-2318-9)
Supplement: Additional file 1: Table S1A. — Validation of three methods of identifying a LTC nursing home stay using Medicaid charges for LTC services as the gold standard, in patients dually eligible for Medicare and Medicaid, stratified by age. Table S1B. Validation of three methods of identifying a LTC nursing home stay using Medicaid charges for LTC services as the gold standard in patients dually eligible for Medicare and Medicaid, stratified by race. Table S1C. Validation of three methods of identifying a LTC nursing home stay using Medicaid charges for LTC services, as the gold standard in patients dually eligible for Medicare and Medicaid, stratified by hospitalization or SNF stay in that year. Table S1D. Validation of three methods of identifying a LTC nursing home stay using Medicaid charges for LTC services as the gold standard in patients dually eligible for Medicare and Medicaid, stratified by gender and location. (DOCX 16 kb) [file 12913_2017_2318_MOESM1_ESM.docx]

**Additional file**

Table S1A: Validation of three methods of identifying a LTC nursing home stay using Medicaid charges for LTC services as the gold standard, in patients dually eligible for Medicare and Medicaid, stratified by age.

|  | Age | | |  |
| --- | --- | --- | --- | --- |
|  | <65 | 65-75 | 75-85 | >=85 |
|  | N=7,566 | N=11,048 | N=18,760 | N=20,924 |
| **Sensitivity (%)** |  |  |  |  |
| 1) Part A and B with E&M charge for NH service | 94.95% | 93.61% | 91.57% | 91.32% |
| 2) Part A and MDS episode of care | 92.82% | 92.15% | 93.05% | 93.67% |
| 3) CMS method: MDS alone | 91.45% | 90.87% | 91.58% | 92.54% |
| **PPV (%)** |  |  |  |  |
| 1) Part A and B with E&M charge for NH service | 62.53% | 70.32% | 70.96% | 69.92% |
| 2) Part A and MDS episode of care | 88.04% | 87.44% | 86.14% | 83.59% |
| 3) CMS method: MDS alone | 81.90% | 81.57% | 81.21% | 80.34% |

Table S1B: Validation of three methods of identifying a LTC nursing home stay using Medicaid charges for LTC services as the gold standard in patients dually eligible for Medicare and Medicaid, stratified by race.

|  | Race/ Ethnicity | | |  |
| --- | --- | --- | --- | --- |
|  | White | Black | Hispanic | Other |
|  | N=37,290 | N=8,490 | N=10,060 | N=2,458 |
| **Sensitivity (%)** |  |  |  |  |
| 1) Part A and B with E&M charge for NH service | 92.01% | 93.17% | 92.79% | 91.86% |
| 2) Part A and MDS episode of care | 93.67% | 91.76% | 92.08% | 92.76% |
| 3) CMS method: MDS alone | 92.40% | 90.21% | 90.71% | 91.01% |
| **PPV (%)** |  |  |  |  |
| 1) Part A and B with E&M charge for NH service | 68.40% | 68.73% | 72.02% | 68.22% |
| 2) Part A and MDS episode of care | 84.60% | 87.08% | 88.35% | 86.99% |
| 3) CMS method: MDS alone | 80.49% | 81.07% | 83.32% | 80.64% |

Table S1C: Validation of three methods of identifying a LTC nursing home stay using Medicaid charges for LTC services, as the gold standard in patients dually eligible for Medicare and Medicaid, stratified by hospitalization or SNF stay in that year.

|  | SNF | | Hospitalization | |
| --- | --- | --- | --- | --- |
|  | No | Yes | No | Yes |
|  | N=43,507 | N=14,791 | N=36,670 | N=21,628 |
| **Sensitivity (%)** |  |  |  |  |
| 1) Part A and B with E&M charge for NH service | 90.95% | 96.28% | 91.04% | 94.44% |
| 2) Part A and MDS episode of care | 94.71% | 88.29% | 95.25% | 89.41% |
| 3) CMS method: MDS alone | 93.01% | 88.14% | 94.46% | 87.22% |
| **PPV (%)** |  |  |  |  |
| 1) Part A and B with E&M charge for NH service | 73.44% | 57.53% | 76.86% | 57.05% |
| 2) Part A and MDS episode of care | 87.14% | 81.79% | 88.39% | 81.63% |
| 3) CMS method: MDS alone | 87.97% | 66.56% | 88.56% | 71.18% |

Table S1D: Validation of three methods of identifying a LTC nursing home stay using Medicaid charges for LTC services as the gold standard in patients dually eligible for Medicare and Medicaid, stratified by gender and location.

|  | Location | | Gender | |
| --- | --- | --- | --- | --- |
|  | Metro | Non-metro | Female | Male |
|  | N=42,461 | N=15,837 | N=40,740 | N=17,558 |
| **Sensitivity (%)** |  |  |  |  |
| 1) Part A and B with E&M charge for NH service | 95.12% | 84.75% | 91.92% | 93.19% |
| 2) Part A and MDS episode of care | 93.90% | 90.87% | 93.10% | 93.03% |
| 3) CMS method: MDS alone | 92.66% | 89.39% | 91.90% | 91.47% |
| **PPV (%)** |  |  |  |  |
| 1) Part A and B with E&M charge for NH service | 68.46% | 71.60% | 69.45% | 68.14% |
| 2) Part A and MDS episode of care | 85.37% | 86.50% | 85.23% | 86.70% |
| 3) CMS method: MDS alone | 80.66% | 82.13% | 81.04% | 81.08% |
